# Supplementary material for: Awareness and Attitudes Regarding Industrial Food Fortification in Mongolia and Harbin
Source: Nutrients. 2019 Jan 19;11(1):201. doi: 10.3390/nu11010201 (PMC6356891; doi:10.3390/nu11010201)
Supplement: Supplementary file 1 [file nutrients-11-00201-s001.pdf]

Awareness and attitudes regarding industrial food fortification in Mongolia and Harbin

Sabri Bromage, Enkhmaa Gonchigsumlaa, Margaret Traeger, Bayarbat Magsar, Qifan Wang, Jorick Bater, Hewei Li and Davaasambuu Ganmaa

Table S1. Survey reproducibility in Mongolia (percent agreement between repeated assessments).

**Survey Question** (% agreement expected by chance alone provided for comparison)	**Rural (n=28)**	**Urban (n=41)**
**Characteristics of study population**		
How much of the food shopping do you usually do for your household? (25%)	36	54 [b]
How many servings of fruit do you typically eat in a day? (25%)	55 [b]	44 [b]
How many servings of vegetables do you typically eat in a day? (25%)	25	28
How much milk do you typically drink in a day? (25%)	56 [b]	65 [b]
Do you consider yourself the right weight, underweight or overweight? (33.3%)	68 [b]	78 [b]
**Awareness of food fortification**		
Are vitamins or minerals sometimes added to foods and drinks by the manufacturer? (33.3%) [a]	61 [b]	37
Does the government mandate the addition of vitamins or minerals to certain foods? (33.3%)	54	38
Which foods or drinks is it mandatory that vitamins or minerals be added to? (33.3%) *	47	49
Last time you bought/consumed a fortified food, how did you know it was fortified? (50%) *	79 [b]	83 [b]
Where on food packaging have you seen a statement regarding added micronutrients? (50%) *	76 [b]	75 [b]
If you wanted to know if a food had added vitamins/minerals, where would you look? (50%) *	77 [b]	69 [b]
**Attitudes toward food fortification**		
Would you prefer that your child(ren) drink vitamin D fortified milk or unfortified milk? (50%)	93 [b]	87 [b]
Do you think others would be receptive to fortification of milk products? (33.3%)	79 [b]	76 [b]
If you found that a food was fortified, would you be more or less likely to buy/consume it? (20%)	43 [b]	39 [b]
How much more would you be willing to pay for fortified food products? (20%)	39	29
Should the government mandate the addition of vitamins and minerals to foods? (33.3%)	46	50
How does knowing the purpose of vitamin D fortification of milk affect your opinion of it? (20%)	50 [b]	55 [b]
What is the strength of your opinion on your previous answer? (25%)	43	53 [b]
Which foods do you buy/consume because they have added vitamins or minerals? (50%) *	67	68

Footnote: [a] Percent agreement differs significantly between rural and urban Mongolia (two-sample test of proportions $p<0.05$). [b] Percent agreement significantly exceeds that expected by chance alone (two-sample test of proportions $p<0.05$). * Percent agreement for this question is calculated as an average over multiple subcategories listed in Tables 2 and 3.

Table S2. Adjusted associations between study site and fortification awareness/attitudes

Survey Question	Rural Mongolia (ref: Urban Mongolia)		Rural Mongolia (ref: Harbin)		Urban Mongolia (ref: Harbin)	
	OR	*p*	*OR*	*p*	*OR*	*p*
**Are vitamins or minerals sometimes added to foods and drinks by the manufacturer? (ref: I'm not sure)**						
Yes	0.38	0.0731	0.16	0.0003	0.42	0.0076
No	0.75	0.6537	0.86	0.8103	1.15	0.747
**Does the government mandate the addition of vitamins or minerals to certain foods? (ref: I'm not sure)**						
Yes	3.09	0.0657	2.06	0.1862	0.67	0.3176
No	0.39	0.0801	0.37	0.0544	0.96	0.9102
**Would you prefer that your child(ren) drink vitamin D fortified milk or unfortified milk? (ref: Unfortified)** [a]						
Fortified	0.46	0.4305	9.94	0.0036	21.59	<.0001
**Do you think others would be receptive to fortification of milk products? (ref: No or I'm not sure)**						
Yes	1.15	0.806	4.07	0.0046	3.55	0.0008
**If you found that a food or drink had added vitamins or minerals, would this make you: (ref: Other** [a]**)**						
More likely to buy or consume it	0.52	0.2668	3.60	0.0149	6.92	<.0001
Depends on the food, drink, vitamin, or mineral	0.38	0.1066	1.16	0.777	3.05	0.0057
**How much more would you be willing to pay for fortified food products? (ref: I would not pay more)**						
5-10%	0.82	0.6911	0.58	0.2577	0.71	0.2954
15-20%	3.03	0.0817	1.71	0.3393	0.57	0.2161
**Should the government mandate the addition of vitamins and minerals to food? (ref: I don't know)**						
Yes	1.78	0.2618	5.09	0.001	2.87	0.0021
No	1.50	0.5492	0.71	0.5677	0.48	0.0767
**Vitamin D may be added to milk to reduce risk of bone deformities in children. Knowing this, would you say: (ref: Other b)**						
Milk fortification should be mandatory	1.66	0.3672	37.46	<.0001	22.59	<.0001
**What is the strength of your opinion on your previous answer? (ref: Moderate or I don't know)**						
Somewhat strong	0.71	0.6342	3.72	0.0269	5.23	0.0007
Very strong	0.78	0.7554	7.69	0.0033	9.84	<.0001

Footnote: Odds ratios and p values are obtained from logistic or multinomial logistic models of the relationship between survey responses (dependent variable) and study site, adjusted for sex, age, age^2, university education, self-reported bodyweight classification, and proportion of food shopping done for the household. [a] Survey response reference category: combination of "Less likely to buy or consume it", "It makes no difference", and "I don't know". [b] Survey response reference category: combination of "Milk fortification should be voluntary", "I'm not sure", and "I don't care". n = 286 nonmissing observations for all models except that indicated by [a] (for which n = 292 nonmissing observations).

Table S3. Adjusted associations between fortification awareness/attitudes and proclivity to purchase or use fortified foods across study sites

Survey Question	Breakfast cereal		Margarine-like spreads		Other dairy products		Milk		Flour		Salt		Noodles		Fruit juice		Flavored or smart water		Other foods	
	OR	*p*	*OR*	*p*	*OR*	*p*	*OR*	*p*	*OR*	*p*	*OR*	*p*	*OR*	*p*	*OR*	*p*	*OR*	*p*	*OR*	*p*
**Are vitamins or minerals sometimes added to foods and drinks by the manufacturer? (ref: I'm not sure)**																				
Yes	1.26	0.529	1.98	0.192	1.70	0.099	0.78	0.571	1.67	0.199	1.95	0.076	2.66	0.022	0.68	0.252	0.34	0.032	0.63	0.542
No	1.09	0.869	3.04	0.062	0.55	0.158	0.92	0.895	1.80	0.242	2.09	0.124	3.86	0.014	0.41	0.075	0.42	0.189	0.29	0.308
**Does the government mandate the addition of vitamins or minerals to certain foods? (ref: I'm not sure)**																				
Yes	1.85	0.261	0.83	0.766	1.18	0.700	2.04	0.27	3.08	0.034	2.16	0.137	1.29	0.667	0.60	0.267	2.55	0.187	4.19	0.190
No	1.86	0.163	1.14	0.812	1.11	0.778	2.25	0.128	1.47	0.403	2.37	0.046	3.45	0.014	1.02	0.957	2.52	0.126	4.00	0.158
**Would you prefer that your child(ren) drink vitamin D fortified milk or unfortified milk? (ref: Unfortified)**																				
Fortified	0.61	0.234	4.32	0.033	2.48	0.019	0.52	0.166	0.62	0.364	0.96	0.933	1.56	0.395	2.28	0.061	0.55	0.234	0.61	0.548
**Do you think others would be receptive to fortification of milk products? (ref: No or I'm not sure)**																				
Yes	1.32	0.500	0.25	0.010	1.40	0.330	0.86	0.755	0.83	0.661	0.62	0.235	0.71	0.452	0.79	0.537	0.27	0.012	4.30	0.140
**If you found that a food or drink had added vitamins or minerals, would this make you: (ref: Other [a])**																				
More likely to buy or consume it	1.25	0.631	6.01	0.005	0.66	0.286	1.16	0.793	4.95	0.002	4.29	0.002	8.80	0.000	1.05	0.917	6.03	0.006	3.50	0.137
Depends on the food, drink, vitamin, or mineral	0.89	0.761	1.65	0.431	0.67	0.231	1.46	0.398	2.86	0.033	2.68	0.026	4.48	0.005	1.27	0.523	5.43	0.002	0.90	0.894
**How much more would you be willing to pay for fortified food products? (ref: I would not pay more)**																				
5-10%	1.85	0.134	1.07	0.908	0.90	0.765	0.99	0.983	0.90	0.784	0.59	0.156	0.56	0.168	0.96	0.911	1.50	0.440	0.37	0.235
15-20%	1.00	0.993	1.68	0.420	1.72	0.196	4.63	0.007	0.65	0.401	0.33	0.030	0.42	0.127	1.70	0.226	1.09	0.905	0.51	0.486
**Should the government mandate the addition of vitamins and minerals to food? (ref: I don't know)**																				
Yes	0.88	0.803	1.78	0.311	0.95	0.901	0.40	0.132	0.49	0.129	0.55	0.175	0.51	0.175	1.18	0.685	1.90	0.354	0.87	0.876
No	1.88	0.148	1.44	0.583	0.85	0.685	0.91	0.851	0.85	0.752	0.59	0.270	0.28	0.022	0.57	0.207	2.34	0.162	0.72	0.727
**Vitamin D may be added to milk to reduce the risk of bone deformities in children. Knowing this, would you say: (ref: Other [b])**																				
Milk fortification should be mandatory	1.46	0.434	0.24	0.012	1.85	0.118	1.88	0.255	0.49	0.109	0.34	0.018	0.29	0.013	1.21	0.637	0.35	0.112	0.36	0.281

Footnote: Odds ratios and p values are obtained from logistic or multinomial logistic models of the relationship between survey responses (dependent variable) and affirmative response to the question "Would you buy or consume this food because it has added vitamins or minerals?", adjusted for study site, sex, age, age^2, university education, self-reported bodyweight classification, proportion of food shopping done for the household, and other 7 survey questions in the table. [a] Survey response reference category: combination of "Less likely to buy or consume it", "It makes no difference", and "I don't know". [b] Survey response reference category: combination of "Milk fortification should be voluntary", "I'm not sure", and "I don't care". n = 286 nonmissing observations for all models.

Document S1: Food Fortification Survey Instrument

(Note: In summarizing results of the survey in Tables 1-3 and S1, some questions and responses have been condensed to save space, and their wording may therefore differ slightly from this instrument.)

1. How much of the food shopping do you usually do for your household?
A. All or most of the shopping
B. About half of the shopping
C. Less than half of the shopping
D. None of the shopping
E. Don't know

2. Would you prefer that your child(ren) drink vitamin D fortified milk or unfortified milk?
A. Fortified
B. Unfortified

3. Do you think other Mongolians would be receptive to fortification of milk products?
A. Yes
B. No

4. Do you think it is true or false, or are you unsure, whether foods and drinks sometimes contain vitamins or minerals that have been added to them by the manufacturer?
A. True
B. False
C. Unsure

5. If you found out that the food or drink you were thinking of buying or consuming did have vitamins or minerals added by the manufacturer, would this make you:
A. More likely to buy or consume it
B. Less likely to buy or consume it
C. It makes no difference
D. It depends on the type of food or drink, or the vitamin or mineral that was added
E. Don't know

6. How much more would you be willing to pay for fortified food products?
A. 5% more
B. 10% more
C. 15% more
D. 20% more
E. I would not pay more

7. For each of the following food or drink categories, are there any food items that you buy or consume because they have added vitamins or minerals? (Multiple responses are acceptable.)

A. Breakfast cereals
B. Margarines or similar spreads
C. Dairy products such as yoghurt or milk
D. Milk substitutes, such as soy or rice milk
E. Flour
F. Salt
G. Pasta
H. Fruit juice
I. Flavored or smart waters such as Vitamin Water, Nutrient Water or G-force
J. Some other type of food or drink (Specify:____________________)
K. None
L. Don't know

8. Thinking about the last time you bought or consumed a food with added vitamins or minerals. How did you know it had vitamins or minerals added by the manufacturer? (Multiple responses are acceptable.)

A. Read it on the pack / container
B. From a TV / newspaper / magazine ad
C. Read it in an article
D. Heard from friends / family
E. Other (Specify:____________________)
F. Don't know
G. I have no bought or consumed a food with added vitamins or minerals

9. Where on the pack did you see it had added vitamins or minerals? (Multiple responses are acceptable.)

A. A statement on the front, such as "added calcium" or something similar
B. On the nutrition information panel
C. In the ingredient list
D. Or somewhere else on the pack (Specify:____________________)
E. Don't know
F. Not applicable

10. If you were buying or consuming a food product and you wanted to see whether it had vitamins or minerals added to it by the manufacturer, where on the pack would you look? (Multiple responses are acceptable.)

A. On the front of the pack, to see if there was a statement such as "added calcium" or something similar
B. On the nutrition information panel
C. In the ingredient list
D. Or somewhere else on the pack (Specify:____________________)
E. Don't know

11. Do you think the government does or does not make it mandatory for food manufacturers to add vitamins or minerals to some types of food?
A. Yes, government does make it mandatory
B. No, government does not make it mandatory
C. Don't know

12. Do you think the government should make it mandatory for food manufactures to add vitamins and minerals to food?
A. Yes, government does make it mandatory
B. No, government does not make it mandatory
C. Don't know

13. Is it true or false or are you unsure if vitamins or minerals must be added to:

	A. True	B. False	C. Unsure
1. Candy	1	2	3
2. Breakfast Cereal	1	2	3
3. Flour	1	2	3
4. Margarine/Butter	1	2	3
5. Fruit Juice	1	2	3
6. Salt	1	2	3
7. Milk/Milk Substitutes	1	2	3

14. The reason milk should have vitamin D added to it is to reduce the risk of children developing bone deformities like rickets. Knowing this, would you say:
A. It should be mandatory for manufacturers to vitamin D to all milk
B. It should be optional for manufacturers to vitamin D to all milk
C. It's difficult to decide either way
D. I don't care either way

15. What is the strength of your opinion on this?
A. Very strong
B. Somewhat strong
C. Or, not at all strong

16. How many servings of vegetables do you usually eat each day? Do not include vegetable juice. (One serving is equal to half a cup of cooked vegetables or legumes, or 1 cup of salad vegetables.)
A. Don't eat any vegetables

B. 1 serving or less per day
C. 2 servings
D. 3 servings
E. 4 servings
F. 5 servings
G. 6 servings or more per day
H. Don't know

17. How many servings of fruit do you usually eat each day? Do not include fruit juice. (One serving is equal to 1 medium fruit, e.g. 1 apple, 2 small fruit eg 2 apricots, or 1 cup of chopped or canned fruit.)
A. Don't eat any fruit
B. 1 servings or less per day
C. 2 servings
D. 3 servings
E. 4 servings
F. 5 servings
G. 6 servings or more per day
H. Don't know

18. How much milk do you drink a day?
A. 250 milliliters
B. 500 milliliters
C. 750 milliliters
D. 1,000 milliliters
F. 1,250 or more milliliters

19. How much milk, on average, does your child(ren) drink a day?
A. 250 milliliters
B. 500 milliliters
C. 750 milliliters
D. 1,000 milliliters
F. 1,250 or more milliliters

20. Do you consider yourself to be about the right weight, underweight or overweight?
A. About the right weight
B. Underweight
C. Overweight
D. Refused

21. Do you consider your children to be about the right weight, underweight, or overweight?
A. About the right weight

B. Underweight
C. Overweight
D. Refused

22. How frequently do you shop for milk?
A. Once a day or more
B. Several times a week
C. Once a week
D. Several times a month
E. Less than once a month
F. Never

23. How frequently do you shop for flour?
A. Once a day or more
B. Several times a week
C. Once a week
D. Several times a month
E. Less than once a month
F. Never

24. How frequently do you shop for food for yourself and your family at each of the following types of venues?

	A. Once a day or more	B. Several times a week	C. Once a week	D. Several times a month	E. Less than once a month	F. Never
1. Large chain grocery store						
2. Large chain store						
3. Locally owned grocery store						
4. Specialty food store						
5. Local market						
6. Local or regional farmer's market						
7. Direct from farm/producer						
8. Internet site of large producer						
9. Internet site of small producer						
10. Catalog						

25. What is the highest educational qualification you have completed?
A. University degree or higher (including postgraduate diploma)

B. Undergraduate diploma or associate diploma
C. Certificate, trade qualification or apprenticeship
D. Highest level of secondary school
E. Did not complete highest level of school
F. Never went to school
G. Still at secondary school
H. Other (Specify:____________________)
I. Refused

26. What is your household's combined monthly income from all sources, before tax, in local currency?
A. Specify:____________________
B. Refused
C. Don't know

27. Last week, did you have a full-time or part-time job of any kind?
A. Yes
B. No

28. If yes, was that:
A. Work for payment or profit
B. Absent on holidays, on paid leave, on strike, or temporarily stood down
C. Unpaid work in a family business
D. Other unpaid work

29. In your main job held last week, what was your occupation? ____________________

30. What are the main tasks you usually perform in that occupation? ____________________

31. Where in Mongolia/China are you from? ____________________

32. What is your sex?
A. Female
B. Male

33. What age group do you belong to?
A. 16 to 17
B. 18 to 19
C. 20 to 24
D. 25 to 29
E. 30 to 34
F. 35 to 39

G. 40 to 44

H. 45 to 49

I. 50 to 54

J. 55 to 59

K. 60 to 64

L. 65 to 69

M. 70 years and over

N. Refused
